# Bovines Harbor a Diverse Array of Vector-Borne Pathogens in Northeast Algeria

**DOI:** 10.3390/pathogens9110883

**Published:** 2020-10-25

**Authors:** Ghania Boularias, Naouelle Azzag, Christelle Gandoin, Corinne Bouillin, Bruno Chomel, Nadia Haddad, Henri-Jean Boulouis

**Affiliations:** 1 Research Laboratory for Local Animal Resources Management (GRAL), National Higher Veterinary School of Algiers, Rue Issad Abbes, El Alia, 16025 Algiers, Algeria; ghania.boularias@gmail.com; 2 UMR BIPAR, National Veterinary School of Alfort, Anses, INRAE, Paris-Est University, 7 Avenue du Général de Gaulle, 94700 Maisons-Alfort, France; christelle.gandoin@vet-alfort.fr (C.G.); corinne.bouillin@vet-alfort.fr (C.B.); nadia.haddad@vet-alfort.fr (N.H.); 3 Department of Population Health and Reproduction, School of Veterinary Medicine, University of California, Davis, CA 95616, USA; bbchomel@ucdavis.edu

**Keywords:** *Anaplasma* spp., *Theileria* spp., *Babesia* spp., hemotropic *Mycoplasma* spp., co-infections, PCR, cattle, Algeria

## Abstract

Arthropod-borne hemoparasites represent a serious health problem in livestock, causing significant production losses. Currently, the evidence of *Anaplasma* spp., *Theileria* spp., *Babesia* spp., and hemotropic *Mycoplasma* spp. in Algeria remains limited to a few scattered geographical regions. In this work, our objectives were to study the prevalence of these vector-borne pathogens and to search other agents not yet described in Algeria as well as the identification of statistical associations with various risk factors in cattle in the northeast of Algeria. Among the 205 cattle blood samples tested by PCR analysis, 42.4% positive results were obtained for at least one pathogen. The overall rates of *Anaplasma* spp., *Theileria*/*Babesia* spp., and *Mycoplasma* spp. in the cattle sampled were respectively 30.7%, 18.5%, and 2.9%; co-infections with multiple species was also detected. *Anaplasma* spp. and *Theileria/Babesia* spp. were detected at a higher rate in cattle under 3 years old, according to univariate analysis. *Anaplasma* spp. DNA was detected more frequently in our sample in cattle living in semi extensive farming. Our study provides additional data about *Anaplasma* spp., *Theileria/Babesia* spp. and reveals for the first time that *Mycoplasma wenyonii* and ‘*Candidatus* Mycoplasma hemobos are present in cattle in Northeast Algeria.

## 1. Introduction

Investigations on the presence and prevalence of bacterial and protozoal pathogenic agents transmitted by arthropods to cattle and the identification of statistical associations with some demographic, breeding, and environmental factors are a prerequisite for a comprehensible explanation of their circulation. These pathogens are unevenly distributed within countries, depending, in particular, on the presence of specific arthropod vectors. Vector-borne diseases are known to cause various symptoms, including a transient mild fever often followed by an alteration in the general health status with hemolytic anemia, anorexia, and abortions, which can lead to death for some animals. These diseases have not only an impact on milk and meat production but on costs of treatment and prevention as well. In addition, apparently asymptomatic or poorly symptomatic infections could have underestimated impacts on cattle production and/or the evolution of coinfections with better-known pathogens [1,2].

Monitoring of arthropod-borne pathogens (ABP) in bovine populations is important in order to predict the risk of infection in given areas. It allows an early establishment of appropriate control measures. Among ABP, three major groups are of particular veterinary importance: *Anaplasma* spp.*, Theileria/Babesia* spp*.,* and hemotropic *Mycoplasma* spp. Climate and other environmental changes are expected to increase the activity and geographic extent of a number of tick species along with the pathogens they carry, consequently increasing the risk of tick-borne diseases in years to come [3].

Currently, there are seven recognized *Anaplasma* species causing infection in cattle*: A. marginale, A. centrale, A. bovis, A. phagocytophilum, A. platys-*like, *A. capra,* and *A. ovis* [4,5]. All of these are tick-borne, obligatory intracellular, gram-negative bacteria that differ in their host cell tropism. *A. marginale*, *A. centrale,* and *A. ovis* colonize erythrocytes, while *A*. *bovis*, *A. phagocytophilum,* and *A. platys* infect monocytes, neutrophils, and platelets respectively [6,7]. *A. marginale* is recognized as the most pathogenic species in cattle [8]. *A. centrale* is a naturally attenuated species that has been used as a vaccine for the control of bovine anaplasmosis in several countries [9]. *A. bovis* infection has been reported as asymptomatic; however, it can cause a variety of clinical signs [10]. *A. phagocytophilum*, *A. platys*, *A. ovis,* and the recently identified *A. capra* are zoonotic species. *A. phagocytophilum* causes tick-borne fever in cattle and *A. platys* induce cyclic thrombocytopenia in dogs [11]. *A. ovis* that primarily infects sheep was detected in humans with symptoms in Cyprus [12]. Recently, *A. capra* has been identified as the causative agent of anaplasmosis in cattle in China [13].

Genera *Theileria* and *Babesia* are widespread tick-borne hemoparasitic pathogens that can induce cattle diseases. *Theileria* multiplies first in lymphocytes and then in erythrocytes, while *Babesia* multiplies exclusively in erythrocytes [14]. Cattle can be infected by a large number of species, however, only a few of them can cause clinical signs. *T. annulata* and *T. parva* are the most pathogenic species [15]. *T*. *mutans* and *T. velifera* are mildly pathogenic even non-pathogenic species [16]. The subclinical infections by *T. orientalis* (previously known as *T. sergenti, T. buffeli,* and *T. orientalis*) were also reported in cattle [17]. Recently, it has been shown that some genotypes have caused outbreaks in cattle in Japan, Korea, China, Australia, and New Zealand [17]. Among bovine *Babesia* parasites, clinical signs were mentioned for *B. bovis, B. bigemina,* and *B. divergens* while only a subclinical infection was described for *B. major* and *B. occultans* [18].

Bovine hemoplasmosis is caused by hemotropic *Mycoplasma* that is an epi-erythrocytic bacteria triggering anemia in infected animals [19]. Two species have been reported in cattle: *M. wenyonii* and ‘*Candidatus* Mycoplasma hemobos [19,20].

In cattle, the occurrence of tick-borne pathogens directly correlates with the distribution of tick vectors in the area. Indeed, *T. annulata* transmitted by *Hyalomma* tick species occurs in the Mediterranean Basin, North-East Africa, Middle East, and South Asia [15], while *T. orientalis* is present in areas where *Haemaphysalis* ticks are abundant [17]. *Babesia* infections are frequent in areas where ticks from the genus *Rhipicephalus (Boophilus*) predominate. This tick is recognized as the vector of *B. bigemina* and *B. bovis,* while the species *Ixodes ricinus* is the main vector for *B. divergens* [14]. Several tick species have been associated with the transmission of *Anaplasma* species, including *Ixodes* spp., *Dermacentor* spp., and *Rhipicephalus* spp. [8,21]. There is no direct evidence that hemotropic *Mycoplasma* is transmitted by ticks [22], whereas some studies have shown that transmission can occur either via blood-sucking flies or lice or iatrogenically by contaminated needles [23].

The objective of our research was to determine the prevalence of pathogenic species belonging to *Anaplasma*, *Theileria*/*Babesia,* and hemotropic *Mycoplasma* as well as co-infection cases and to identify statistical associations with some risk factors in cattle. For that, a cross-sectional study, based on PCR analysis, was carried out on blood samples collected from cattle in north-eastern Algeria.

## 2. Results

### 2.1. Molecular Detection of Anaplasma, Theileria/Babesia, and Hemotropic Mycoplasma Species

PCR-based investigation of 205 cattle showed a total infection rate of 42.4% (87/205) including both single and coinfection cases. Single, double, and triple infections were identified at the respective rates of (57/205) (27.8%), 27/205 (13.2%), and 3/205 (1.4%) (Table 1).

**Figure 1:**
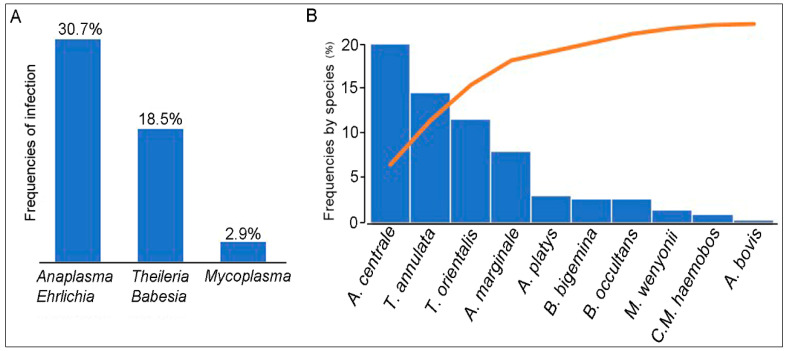
(**A**) Individual rate of *Anaplasma/Ehrlichia* spp., *Theileria/Babesia* spp., and *Mycoplasma* spp. of 205 cattle tested by PCR. (**B**) The figure shows in decreasing order the frequencies of each detected species.

The overall frequency of *Ehrlichia/Anaplasma* spp., *Theileria/Babesia* spp., and *Mycoplasma* spp. was respectively 30.7% (63/205), 18.5% (38/205), and 2.9% (6/205) (Figure 1). Among the *Ehrlichia/Anaplasma* spp. detected, *A. centrale* (37/205, 18%) was the most frequently recorded species, followed by *A. marginale* (15/205, 7.3%) and *A. bovis* (1/205, 0.5%), while the DNA of *A. phagocytophilum* and *A. capra* were not detected. The 16S PCR products of *A. bovis* were sequenced and BLAST analysis confirmed 94.84% identity with *A. bovis* 16S gene sequence analyzed (GenBank accession number KX450273.1). The sequencing of *Ehrlichia/Anaplasma*-positive PCR products that were negative for the presence of the five *Anaplasma* species mentioned above, revealed 2,9% (6/205) positive samples for *A. platys* and 2.9% (6/205) for uncultured *Anaplasma* sp. The sequencing of these products showed A 100% identity with *A. platys* (GenBank accession number MK386768.1) or uncultured *Anaplasma* sp. clone AMCRO1 (MN187218.1) respectively. Thirteen samples randomly selected among the 38 cattle (18.5%) positive samples for *Theileria/Babesia* DNA were sequenced. Five, four, and two samples were identified as *T. annulata*, *T. orientalis,* and *Theileria* sp. respectively, while a single sample was positive for *B. occultans* and *B. bigemina*. All positive PCR products for *T. annulata* shared 97.6% sequence identity with GenBank acc. no. MF287924.1, for *T. orientalis*, 96.7% with GenBank no. MN187008.1, for *Theileria* sp., 99.4% with GenBank no. AJ616717.1, for *B. occultans,* 91.3% with GenBank no. MK421149.1, and for *B. bigemina,* 83.3% with GenBank no. MK732475.1. Among the six samples that were positive for *Mycoplasma* spp. DNA, three samples were positive for *M. wenyonii* and two for ‘*Candidatus* Mycoplasma hemobos. The DNA sequences of the two identified hemotropic *Mycoplasma* species showed 98.9% and 98.5% identity with those from ‘*Candidatus* Mycoplasma hemobos and *M. wenyonii* available on GenBank: MG948633.1 and MG948626.1 respectively.

### 2.2. Variability of Vector-Borne Pathogens Infection Associated to Different Intrinsic and Environmental Factors

No statistically significant association was observed between the level of *Anaplasma* and hemotropic *Mycoplasma* infection and sex of cattle (*p* (χ^2^) > 0.05), except for *Theileria/Babesia*, for which a significant difference (*p* (χ^2^) = 0.04) between males (8/24, 33.3%) and females (30/181, 16.5%) was detected. Cattle under 3 years of age were more frequently infected with *Anaplasma* spp. (31/75, 41%) (*p* (χ^2^)= 0.02) and *Theileria/Babesia* (20/75, 26.6%) (*p* (χ^2^) = 0.02) than cattle ≥3 years old (32/130, 24.6%, 18/130, 13.8%). For hemotropic *Mycoplasma*, no statistically significant difference in frequency (*p* (χ^2^)= 0.49) based on the age of cattle was detected (Table 2). Regarding the farming system, the frequency of *Anaplasma* spp. infection (59/169, 34.9%) in animals living in semi extensive/extensive farms was significantly higher (*p* (χ^2^) = 0.005) than cattle raised in intensive production systems (4/36, 11.1%). For *Theileria/Babesia* and hemotropic *Mycoplasma* infection, the difference between the farming systems was not statistically significant (*p* (χ^2^) > 0.05) (Table 2). A significant difference in prevalence (*p* (χ^2^) = 0.004) between cattle infested with ticks (42/105, 40%) and tick-free cattle (21/100, 21%) was reported for *Ehrlichia/Anaplasma* spp. only. For *Theileria/Babesia* spp. and *Mycoplasma* spp., no significant difference was observed (*p* (χ^2^) > 0.05) according to the presence or absence of ticks on the animals. Finally, no significant association was observed between the overall prevalence of tested pathogens and the parasitic load of ticks on the animal (Table 2).

## 3. Discussion

We carried out a descriptive survey for the determination of infection rates for *Ehrlichia/ Anaplasma, Theileria/Babesia,* and hemotropic *Mycoplasma* species in blood samples from bovine collected in north-eastern Algeria, based on DNA detection. While some information concerning the occurrence of bovine anaplasmosis and piroplasmosis had already been reported in Algeria [24,25,26], the presence of cattle hemoplasmosis (*Mycoplasma* spp.) had never been documented on the African continent. 

This study was carried out in eight municipalities with a cattle population of 44,000 heads, which corresponds approximately to 40% of the cattle population of the region studied. The low representativity of our sample can be explained by the difficulties encountered in the field, i.e., a very limited access to farms located in isolated and mountainous regions, the difficulty to contain animals that are not used to having frequent contact with humans, and the refusal to cooperate by some breeders. All these factors contributed to the selection bias induced and prevented us from inferring the results to the whole region. Our study reported that 42.4% of cattle sampled were infected with at least one of the tested vector-borne agents. The high prevalence of these tick-borne pathogens has also been recorded in other countries, such as Ethiopia, China, and Russia [1,27,28]. Despite the lack of representativity, our results support an important tick infestation and a significant circulation of tick-borne pathogens in the surveyed region.

Four species of *Anaplasma* were detected*,* mainly *A. centrale* and *A. marginale,* whereas *A. bovis* had a low prevalence. These results are in accordance with those of two previous studies carried out in Algeria and Tunisia [25,29]. *A. platys*, the agent of canine infectious cyclic thrombocytopenia, was detected in 2.9% of the tested cattle. In other studies*,* a strain genetically closely related to *A. platys* called *A. platys-*like was detected in ruminants [24,30,31]. However, our primers were not specific for *Anaplasma platys-*like. Therefore, *A. platys* identified in the present study were likely similar to the previously described strains. *A. phagocytophilum* and *A. capra* that were not identified in our study. The transmission of *A. phagocytophilum* is associated with ticks belonging to the *Ixodes* genus [4]. The population density of *I. ricinus* in North Africa is mainly restricted to cooler and more humid areas (rainfall of more than 800 mm per year) of the Mediterranean climatic region, observed in the Atlas mountains [32]. In our study, the *I. ricinus* tick percentage was only 2.1%, which could explain the absence of *A. phagocytophilum* in the investigated area. The zoonotic *A. capra* species has mainly been described in China [13,33]. As this species has only recently been described, very little data exist in the literature. 

The target sequence in the 16S RNA gene of *Theileria/Babesia* spp. was amplified in 18.5% of cattle tested. *T. annulata* was the major species identified (5/13). This species has also been reported as a common pathogen in Tunisia, Spain, Portugal, and Turkey [34,35,36,37]. The detection rate of *T. orientalis* was not negligible either, as 4 out of 13 sequenced specimens tested positive. The frequent occurrence of this species has mostly been associated with cattle from Asia [28,38]. This *Theileria* species do not harbor high pathogenicity for cattle. However, some outbreaks with severe clinical symptoms have been reported in livestock in Japan, Korea, China, Australia, and New Zealand [17]. *B. bigemina* and *B. occultans* DNA were identified in two cattle among the 13 samples sequenced. These two species are transmitted by *Rhipicephalus annulatus* and *Hyalomma marginatum* respectively, both known as the most abundant ticks in North Africa [34,39].

Prior to our study, there was no evidence of hemotropic *Mycoplasma* infection in cattle in Algeria. Therefore, the identification of *M. wenyonii* and ‘*Candidatus* Mycoplasma hemobos in 2.9% of asymptomatic cattle represents the first report of these pathogens in Algeria. In France, *M. wenyonii* was detected in cows with clinical symptoms [40]. The epidemiology of bovine hemoplasmosis is poorly understood. Some studies suggest that ticks could represent biological vectors, while, according to other studies, flies, lice, and fleas could ensure a mechanical transmission of this pathogen [41]. Hemotropic *Mycoplasma*-positive cattle in this study were asymptomatic, which is in agreement with subclinical infections reported by other groups, suggesting that these cattle could be chronic carriers and sources of infection for hemotropic *Mycoplasma*-negative cattle, newly introduced on farms [41,42]. As seen for *Anaplasma* or *Theileria*/*Babesia* infections, this subclinical form can persist for a long period of time after the onset of infection and the affected animals act as reservoirs [21,43].

Cases of co-infection with *Anaplasma* spp. and *Theileria/Babesia* spp. were frequently observed in our study. The same circumstances were described by other groups in China (*Anaplasma* spp., *Theileria* spp. and *Babesia* spp.) and in Russia (*A. marginale, Theileria* spp.) [1,28]. In Japan, authors showed that cattle co-infected with *T. orienatlis* and *Mycoplasma* spp. tend to resist infections by other pathogens, as the degree of anemia observed in co-infected animals was significantly milder than in those infected only with *T. orientalis*. Although the exact mechanism of this phenomenon is unknown, it is possible that the proliferation of *T. orientalis* is also inhibited by the immune response raised against hemoplasma and/or by several other mechanisms [44]. Further studies are needed to evaluate the host response to co-infections. Nevertheless, it appears that complex clinical manifestations due to a high frequency of co-infections influence the duration of infection and the intensity of the symptoms, which subsequently affects the effective control of diseases [1].

The association of a higher rate of infection with *Theileria/Babesia* spp. in male cattle (*p* (*χ*^2^) = 0.04) suggests that animal gender could play a role in the receptivity to these pathogens, as reported by Zhou et al. [28]. In addition, the higher *Anaplasma* spp., *Theileria/Babesia* spp., and hemotropic *Mycoplasma* spp. infection rates in cattle younger than 3 years may be associated with repeated exposures of cattle to these pathogens, allowing cattle to develop protective immunity. In general, a higher frequency for the pathogens investigated was observed in cattle living in semi extensive and extensive livestock (46.7%, *p*(*χ*^2^) = 0.007). There is no doubt that semi and extensive farming potentially increased the risk for cattle to be exposed to tick bites, the probability of tick infection being increased by their proximity with infected wild animals, especially reservoir species.

## 4. Materials and Methods

### 4.1. Ethical Statement 

The sample collection was authorized by the National Veterinary School of Algiers, Algeria, and the agreement of the Veterinary Services Department of the Wilaya of Tizi-Ouzou, Algeria. All cattle were sampled according to Algerian regulations. Blood collection was performed under owners’ presence and standard techniques for collecting blood samples were used, respecting animal welfare.

### 4.2. Sampling and DNA Extraction

**Figure 2:**
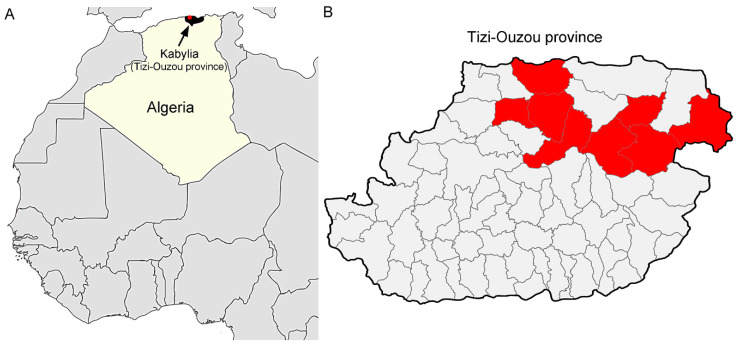
Map of Tizi-Ouzou city (Algeria); (**A**) geographical location of the sample collection; (**B**) elements colored in red correspond to the 8 municipalities selected.

The study was conducted in the Tizi-Ouzou region, a city located in the Northeast of Algeria. The total number of cattle is 110,000 heads, spread over 66 municipalities (data from the Tizi-Ouzou Agricultural Services). For the sampling plan, we proceeded as follows: out of a total of 66 municipalities, 8 were selected, which corresponded to 40% (44, 000 heads) of the bovine livestock and 12.1% of the region studied (Figure 2). This selection was established according to the possibilities of access to the farms. Then, 205 cattle apparently healthy were selected from 35 farms as follows: in each farm, a maximum of 10 cattle was randomly sampled from herds of 10 or more. For herds of less than 10 cattle, all animals were sampled. Blood samples were collected from the caudal vein of cattle between May 2015 and November 2017. A standardized questionnaire was used to obtain information regarding farm management practices and possible risk factors associated with infection with ABP. Information about age and sex was also recorded. For data analysis, two age groups were constituted to compare animals less than 3 years old to older animals. The 3 years cut off was based on the age at first calving. The presence of ticks was recorded; identification was carried out to the genus. The parasitic load is the total number of ticks collected from the sampled cattle (Table 2). A total of 810 ixodid ticks were collected from 105 cattle and belonged to 3 different genera after morphological identification using taxonomic keys developed by Walker et al. [32]: *Rhipicephalus* (n = 491), *Hyalomma* (n = 302), and *Ixodes* (n = 17). The Table S1 contains the data of each individual animal; date of blood sampled, Sex, age, farming system, number and genera of collected ticks and identified pathogens.

DNA was extracted from whole blood samples using a 200 μL EDTA Nucleospin Blood Quickpure kit (NucleoSpin®, Macherey-Nagel, Düren, Germany) according to the manufacturer’s instructions. DNA samples were stored at −20 °C until analyzed by PCR.

### 4.3. Molecular Detection of Ehrlichia/Anaplasma, Theileria/Babesia, and Hemotropic Mycoplasma species 

Extracted DNAs were used as templates for initial standard PCR targeting the 16S RNA gene (Table 3) to detect *Ehrlichia/Anaplasma* spp. [45], *Theileria/Babesia* spp. [46], and *Mycoplasma* spp. [47]. The samples that were positive for *Ehrlichia/Anaplasma* spp. were further investigated for the presence of *A. marginale, A. centrale, A. bovis, A. capra,* and *A. phagocytophilum* DNA. The presence of *A*. *marginale* DNA was investigated by amplifying the gene encoding the major surface protein 1 (*msp1*, Table 3). For the detection of *A. centrale*, *A. bovis,* and *A. capra* DNA, PCR tests were performed with primers for the genes encoding 16S RNA (Table 3). Standard 25 μL volume PCRs containing 5 μL of DNA template were performed using Taq polymerase (Takara Ex Taq, Dalian, China). PCR products were visualized in 2% agarose gel, containing ethidium bromide. *A. phagocytophilum, Babesia caballi* and *Mycoplasma haemofelis* DNAs were used as positive controls for the detection of *Ehrlichia/Anaplasma* spp., *Theileria/Babesia* spp., and *Mycoplasma* spp., respectively. To monitor the occurrence of false-positive PCR results, negative controls (20 μL of reaction mixture + 5 μL of ultrapure water) were included for each amplification. In order to minimize contaminations, the DNA extraction, the reagent set-up, the DNA addition, the PCR, and the sample analysis were performed in four separate rooms. 

Detection of *A. phagocytophilum* was performed using primers for the *msp2* gene (Table 3). The real-time PCR assay for the detection of *A. phagocytophilum* was carried out and the analysis of the results was performed using Light Cycler® 480 Software Version 1.5.1. (Roche Applied Science, Penzberg, Germany). For the *Ehrlichia/Anaplasma-*positive DNA samples that were found to be negative for five *Anaplasma* species, a nested PCR test targeting the *Ehrlichia* 16S rRNA gene (Table 3) was performed, followed by sequencing (Eurofins, Ivry-sur-Seine, France, https://Cochin.eurofins.com) of the amplicon. To detect the *Theileria/Babesia* and *Mycoplasma* species*,* a PCR test targeting the 16S rRNA gene (Table 3) was carried out and the positive PCR products were sequenced (Eurofins). The obtained sequences were analyzed using BioEdit and blasted against online available nucleotide databases in NCBI (https://www.ncbi.nlm.nih.gov). 

### 4.4. Statistical Analysis

The Pearson Chi-square (*χ*^2^) for univariate analyses was calculated using SPSS statistics 20 software (IBM SPSS Statistics 20, France) to assess the association of the frequencies of pathogens with demographic and environmental factors. *p* values ≤0.05 were evaluated as statistically significant.

## 5. Conclusions

In conclusion, this study revealed the circulation of at least ten species of vector-borne pathogens in cattle sampled in the Northeast of Algeria with the first detection of *M*. *wenyonii* and ‘*Candidatus* Mycoplasma hemobos. Several co-infections were noticed. Such cases could have a clinical impact, which affects the effective surveillance and control programs of these diseases. Although our sample was not representative, the diversity of the detected agents and the high frequency for some of them suggest a great abundance of vector-borne agents in the area studied. These results highlight the need for effective control measures to prevent the transmission of tick-borne pathogens to cattle in Algeria. 

## Figures and Tables

**Table 1:** Distribution of single, dual, and triple infection by different species of tick-borne pathogens detected in cattle blood samples.

	Species	Number of Cases	Total	Frequency (%)
**Single** **infection**	*A. centrale*	14		
	*A. marginale*	2		
	*A.* *bovis*	1		
	*A.* *platys*	6	57/205	27.8
	*Anaplasma* sp.	3		
	*M. wenyonii*	1		
	*‘Candidatus* M. haemobos’	1		
	*Mycoplasma* sp.	1		
	*Anaplasma/Ehrlichia* spp.	7		
	*Theileria/Babesia* spp.	21		
**Dual inf** **ection**	*A. centrale, B. occultans*	1		
	*A. centrale,* T*. orientalis*	2		
	*A. centrale, Theileria/Babesia* spp.	4		
	*A.* *centrale, M. wenyonii*	1		
	*A. centrale, ‘Candidatus* M. haemobos’	1	27/205	13.2
	*A. centrale, A. marginale*	11		
	Uncultured *Anaplasma* sp*., Theileria* sp.	1		
	Uncultured *Anaplasma* sp*., T. orientalis*	1		
	Uncultured *Anaplasma* sp*., Theileria/Babesia* spp.	1		
	*Anaplasma/Ehrlichia* spp.*, Theileria/Babesia* spp.	4		
**Trip** **le infection**	*A. centrale* *, A. marginale, T. annulata*	1		
	*A. centrale, A. marginale, T. orientalis*	1	3/205	1.4
	*A. centrale* *, T. annulata, M. wenyonii*	1		
**Overall**		**87**	**87/205**	**42.4**

**Table 2:** Variability of infection by *Ehrlichia/Anaplasma*, *Theileria/Babesia,* and hemotropic *Mycoplasma* genera associated with gender, age, farming system, and tick infestation in cattle from the studied region.

Cattle Group	Category	Number of Cattle	PCR Results							
			Overall Infection	*p* (*χ*^2^) Value	*Ehrlichia/* *Anaplasma*	*p* (*χ*^2^) Value	*Theileria/* *Babesia*	*p* (*χ*^2^) Value	Hemotropic *Mycoplasma*	*p* (*χ*^2^) Value
Gender	Male	24	12 (50%)	0.4	7 (29.1%)	0.8	8 (33.3%)	0.04	2 (8.3%)	0.09
	Female	181	75 (41.4%)		56 (31%)		30 (16.5%)		4 (2.2%)	
Age (year)	<3 years	75	41 (54.6%)	0.007	31 (41%)	0.02	20 (26.6%)	0.02	3 (4%)	0.49
	≥3 years	130	46 (35.3%)		32 (24.6%)		18 (13.8%)		3 (2.3%)	
Farming system	Intensive	36	8 (22.2%)	0.007	4 (11.1%)	0.005	5 (13.8%)	0.42	0	0.24
	Semi/extensive	169	79 (46.7%)		59 (34.9%)		33 (19.5%)		6 (3.5%)	
Ticks	Present	105	50 (47.6%)	0.14	42 (40%)	0.004	20 (19%)	0.82	4 (3.8%)	0.43
	Absent	100	37 (37%)		21 (21%)		18 (18%)		2 (2%)	
Ticks load	<10	80	36 (45%)	0.37	30 (37.8%)	0.82	14 (17.5%)	0.80	3 (3.7%)	0.80
	10–20	18	9 (50%)		8 (42.4%)		4 (31.5%)		1 (5.5%)	
	>20	7	5 (71.4%)		4 (57.1%)		2 (28.5%)		0	

**Table 3:** List of pathogens, target genes, name and sequences of primers/probes used in this study, hybridization temperature (T °C), and length of fragments (pb) amplified by PCR.

Pathogen	Target Gene	Primer Name	Sequence (5’-3’)	Hybridization (T °C)	Length (pb)	Reference
*Ehrlichia/* *Anaplasma*	*RNA 16S*	EHR1 16S F	GGTACCYACAGAAGAAGTCC	52	346	[45]
		EHR1 16S R	TAGCACTCATCGTTTACAGC			
Nested PCR *Ehrlichia/* *Anaplasma*	*RNA 16S*	EHR1 F	GAACGAACGCTGGCGGCAAGC	60	693	[48]
		EHR2 R	AGTA(T/C)CG(A/G)ACCAGATAGCCGC			
		EHR3 F	TGCATAGGAATCTACCTAGTAG	55	592	
		EHR2 R	AGTA(T/C)CG(A/G)ACCAGATAGCCGC			
qPCR *A. phagocytophilum*	*msp2*	APH F	ATG GAA GGT AGT GTT GGT TAT GGT ATT	60	77	[49]
		APH R	TTG GTC TTG AAG CGC TCG TA			
		APH P	TGG TGC CAG GGT TGA GCT TGA GAT TG			
*A. marginale*	*msp1*	Msp1 a F	TGTGCTTATGGCAGACATTTCC	55	1224	[50]
		Msp1 a R	AAACCTTGTAGCCCAACTTATCC			
*A*. *centrale*	*RNA 16S*	AC1f	CTGCTTTTAATACTGCAGGACTA	55	426	[51]
		AC1r	ATGCAGCACCTGTGAGGT			
*A. bovis*	*RNA 16S*	AB1 F	CTCGTAGCTTGCTATGAGAAC	55	551	
		AB1 R	TCTCCCGGCTCCAGTCTG			
*A. capra*	*RNA 16S*	*A. capra* F	GCAAGTCGAACGGACCAAATCTGT	58	1261	[52]
		*A. capra* R	CCACGATTACTAGCGATTCCGACTTC			
*Mycoplasma*	*RNA 16S*	GP03 F	GGGAGCAAACA GGATTAGATA	55	280	[47]
		MGSO R	TGCACCATCTGTCACTCTGTTAACCTC			
*Theileria/Babesia*	*RNA 16S*	RLB-F	GAGGTAGTGACAAGAAATAACAATA	50	502	[46]
		RLB-R	TCTTCGATCCCCTAACTTTC			

## Supplementary Materials

The following are available online at https://www.mdpi.com/2076-0817/9/11/883/s1, Table S1: This table contains the data of each individual animal; date of blood sampled, Sex, age, farming system, number and genera of collected ticks and identified pathogens.
