# Supplementary material for: Bovines Harbor a Diverse Array of Vector-Borne Pathogens in Northeast Algeria
Source: Pathogens. 2020 Oct 25;9(11):883. doi: 10.3390/pathogens9110883 (PMC7692033; doi:10.3390/pathogens9110883)
Supplement: Supplementary file 1 [file pathogens-09-00883-s001.pdf]

Table S1. This table contains the data of each individual animal; date of blood sampled, Sex, age, farming system, number and genera of collected ticks and identified pathogens.

	Intrinsic and environnement factor						Ticks genera			PCR results										
N° Cattle	Date	Sex	Age (year)	Farming system	N° ticks	ticks load	*Rh*	*Hy*	*Ix*	*Ehrlichia /Anaplasma* Spp.	*Anaplasma* sp.	*A.p*	*A. m*	*A c*	*A. b*	*Mycoplasma* Spp.	*M.w*	C.M.h	*Theileria /Babesia* spp.	*Theileria /Babesia* sequencing
1	09/05/2015	F	>3	S	0	0	0	0	0	N	N	N	N	N	N	N	N	N	N	N
2	09/05/2015	F	<3	S	0	0	0	0	0	N	N	N	N	N	N	N	N	N	P	spp.
3	09/05/2015	F	<3	S	7	<10	7	0	0	N	N	N	N	N	N	N	N	N	N	N
4	09/05/2015	F	>3	S	0	0	0	0	0	N	N	N	N	N	N	N	N	N	N	N
5	09/05/2015	F	>3	S	0	0	0	0	0	N	N	N	N	N	N	N	N	N	N	N
6	10/05/2015	F	>3	S	0	0	0	0	0	N	N	N	N	N	N	N	N	N	N	N
7	10/05/2015	F	>3	S	3	<10	0	3	0	P	N	N	N	P	N	N	N	N	P	spp.
8	10/05/2015	F	>3	S	8	<10	0	8	0	N	N	N	N	N	N	N	N	N	N	N
9	15/05/2015	F	<3	S	1	<10	1	0	0	P	N	N	P	P	N	N	N	N	N	N
10	15/05/2015	M	<3	S	35	>20	20	15	0	P	N	N	N	P	N	N	N	N	P	*B. occultans*
11	15/05/2015	F	>3	S	1	<10	0	1	0	N	N	N	N	N	N	N	N	N	N	N
12	15/05/2015	F	>3	S	0	0	0	0	0	P	P	N	N	N	N	N	N	N	N	N
13	15/05/2015	F	>3	S	45	>20	20	25	0	N	N	N	N	N	N	N	N	N	N	N
14	15/05/2015	F	<3	S	23	>20	19	4	0	P	N	N	N	P	N	N	N	N	N	N
15	15/05/2015	F	>3	S	2	<10	2	0	0	P	N	N	N	P	N	N	N	N	N	N
16	15/05/2015	F	>3	S	2	<10	1	1	0	N	N	N	N	N	N	N	N	N	N	N
17	15/05/2015	M	<3	S	1	<10	0	1	0	P	N	N	N	P	N	P	P	N	P	*T. annulata*
18	15/05/2015	M	<3	S	14	10_20	12	2	0	N	N	N	N	N	N	N	N	N	P	*B. bigemina*
19	17/05/2015	F	>3	S	4	<10	2	2	0	P	N	N	N	N	N	N	N	N	N	N
20	17/05/2015	F	>3	S	11	10_20	10	1	0	P	N	N	N	N	N	N	N	N	N	N
21	20/05/2015	F	<3	S	0	0	0	0	0	N	N	N	N	N	N	N	N	N	N	N
22	20/05/2015	F	>3	S	0	0	0	0	0	N	N	N	N	N	N	N	N	N	N	N
23	20/05/2015	M	<3	S	0	0	0	0	0	N	N	N	N	N	N	N	N	N	N	N

24	26/05/2015	F	<3	S	6	<10	0	6	0	P	N	N	N	N	N	N	N	N	P	*T. annulata*
25	26/05/2015	F	<3	S	5	<10	0	5	0	P	N	N	P	P	N	N	N	N	P	*T. orientalis*
26	26/05/2015	F	>3	S	0	0	0	0	0	N	N	N	N	N	N	N	N	N	N	N
27	26/05/2015	F	>3	S	9	<10	7	2	0	N	N	N	N	N	N	N	N	N	N	N
28	26/05/2015	F	>3	S	7	<10	0	7	0	P	N	N	N	P	N	N	N	N	P	spp.
29	26/05/2015	F	>3	S	18	10_20	3	13	2	P	N	N	N	P	N	N	N	N	P	*T. orientalis*
30	30/05/2015	F	>3	I	0	0	0	0	0	N	N	N	N	N	N	N	N	N	N	N
31	30/05/2015	F	<3	I	0	0	0	0	0	N	N	N	N	N	N	N	N	N	N	N
32	31/05/2015	F	<3	I	0	0	0	0	0	P	N	N	N	P	N	N	N	N	P	*T. orientalis*
33	01/06/2015	F	>3	I	0	0	0	0	0	N	N	N	N	N	N	N	N	N	N	N
34	02/06/2015	F	>3	I	0	0	0	0	0	N	N	N	N	N	N	N	N	N	N	N
35	06/06/2015	F	>3	S	22	>20	14	8	0	N	N	N	N	N	N	N	N	N	P	spp.
36	06/06/2015	M	>3	S	17	10_20	15	2	0	P	N	N	N	N	N	N	N	N	P	spp.
37	06/06/2015	M	<3	S	22	>20	22	0	0	P	N	N	N	P	N	N	N	N	N	N
38	06/06/2015	M	<3	S	3	<10	0	3	0	N	N	N	N	N	N	N	N	N	N	N
39	21/06/2015	F	<3	S	8	<10	8	0	0	N	N	N	N	N	N	N	N	N	N	N
40	21/06/2015	F	>3	S	7	<10	7	0	0	N	N	N	N	N	N	N	N	N	N	N
41	22/06/2015	F	>3	S	0	0	0	0	0	N	N	N	N	N	N	N	N	N	N	N
42	22/06/2015	F	<3	S	0	0	0	0	0	N	N	N	N	N	N	N	N	N	N	N
43	23/06/2015	F	<3	S	0	0	0	0	0	N	N	N	N	N	N	N	N	N	N	N
44	23/06/2015	F	>3	S	0	0	0	0	0	N	N	N	N	N	N	N	N	N	N	N
45	23/06/2015	F	<3	S	0	0	0	0	0	P	N	N	P	P	N	N	N	N	N	N
46	23/06/2015	F	>3	S	4	<10	4	0	0	P	N	N	P	P	N	N	N	N	N	N
47	23/06/2015	M	<3	S	0	0	0	0	0	N	N	N	N	N	N	N	N	N	P	spp.
48	23/06/2015	F	>3	S	6	<10	6	0	0	N	N	N	N	N	N	N	N	N	N	N
49	23/06/2015	F	>3	S	5	<10	5	0	0	N	N	N	N	N	N	N	N	N	N	N
50	26/06/2015	F	<3	S	0	0	0	0	0	N	N	N	N	N	N	N	N	N	N	N
51	26/06/2015	F	>3	S	0	0	0	0	0	N	N	N	N	N	N	N	N	N	N	N
52	26/06/2015	F	>3	S	0	0	0	0	0	N	N	N	N	N	N	N	N	N	N	N
53	27/06/2015	F	>3	S	5	<10	4	1	0	P	N	N	N	N	N	N	N	N	N	N

54	27/06/2015	F	<3	S	12	10_20	11	1	0	P	N	N	N	P	N	N	N	N	N	N
55	27/06/2015	F	<3	S	15	10_20	15	0	0	P	N	N	P	P	N	N	N	N	N	N
56	27/06/2015	F	>3	S	0	0	0	0	0	P	N	N	P	N	N	N	N	N	N	N
57	27/06/2015	M	<3	S	0	0	0	0	0	N	N	N	N	N	N	P	P	N	N	N
58	30/06/2015	F	<3	S	19	10_20	12	7	0	P	P	N	N	N	N	N	N	N	P	*T. orientalis*
59	30/06/2015	F	<3	S	9	<10	8	1	0	P	N	N	N	N	N	N	N	N	P	spp.
60	30/06/2015	F	>3	S	14	10_20	10	4	0	P	N	N	N	P	N	P	P	N	N	N
61	30/06/2015	M	>3	S	6	<10	4	2	0	P	P	N	N	N	N	N	N	N	N	N
62	30/06/2015	F	>3	S	0	0	0	0	0	N	N	N	N	N	N	N	N	N	N	N
63	01/07/2015	F	<3	S	0	0	0	0	0	P	N	N	N	P	N	P	N	P	N	N
64	01/07/2015	F	>3	S	4	<10	1	3	0	P	N	N	N	P	N	N	N	N	N	N
65	01/07/2015	M	<3	S	8	<10	6	2	0	P	P	N	N	N	N	N	N	N	P	*Theileria* sp.
66	01/07/2015	F	<3	S	4	<10	4	0	0	P	N	N	P	P	N	N	N	N	P	*T. annulata*
67	01/07/2015	F	<3	S	0	0	0	0	0	N	N	N	N	N	N	N	N	N	P	spp.
68	01/07/2015	F	>3	S	3	<10	3	0	0	P	N	N	P	P	N	N	N	N	N	N
69	01/07/2015	F	>3	S	12	10_20	9	3	0	P	N	N	N	N	P	N	N	N	N	N
70	01/07/2015	F	<3	S	7	<10	3	4	0	P	N	N	N	P	N	N	N	N	P	spp.
71	04/07/2015	F	>3	I	0	0	0	0	0	N	N	N	N	N	N	N	N	N	N	N
72	04/07/2015	F	<3	I	0	0	0	0	0	N	N	N	N	N	N	N	N	N	N	N
73	04/07/2015	F	>3	I	0	0	0	0	0	N	N	N	N	N	N	N	N	N	N	N
74	04/07/2015	M	<3	I	0	0	0	0	0	N	N	N	N	N	N	N	N	N	N	N
75	04/07/2015	F	<3	I	0	0	0	0	0	N	N	N	N	N	N	N	N	N	N	N
76	04/07/2015	F	>3	I	0	0	0	0	0	N	N	N	N	N	N	N	N	N	N	N
77	04/07/2015	F	>3	S	1	<10	1	0	0	N	N	N	N	N	N	N	N	N	P	spp.
78	04/07/2015	F	<3	S	9	<10	8	1	0	P	N	N	N	N	N	N	N	N	N	N
79	04/07/2015	F	>3	S	1	<10	1	0	0	P	N	N	N	P	N	N	N	N	N	N
80	05/07/2015	F	<3	S	0	0	0	0	0	N	N	N	N	N	N	N	N	N	N	N
81	05/07/2015	F	<3	S	0	0	0	0	0	N	N	N	N	N	N	N	N	N	N	N
82	05/07/2015	F	>3	S	0	0	0	0	0	N	N	N	N	N	N	N	N	N	N	N
83	05/07/2015	F	<3	S	0	0	0	0	0	N	N	N	N	N	N	N	N	N	P	*T. annulata*

84	05/07/2015	F	>3	S	0	0	0	0	0	P	N	N	N	N	N	N	N	N	N	N
85	14/07/2015	F	>3	S	0	0	0	0	0	N	N	N	N	N	N	N	N	N	N	N
86	28/07/2015	F	>3	S	5	<10	0	5	0	P	N	N	N	P	N	N	N	N	P	spp.
87	28/07/2015	F	>3	S	9	<10	0	9	0	N	N	N	N	N	N	N	N	N	N	N
88	13/08/2015	F	<3	S	5	<10	2	3	0	P	N	P	N	N	N	N	N	N	N	N
89	13/08/2015	F	>3	S	0	0	0	0	0	P	N	N	N	N	N	N	N	N	P	spp.
90	13/08/2015	F	<3	S	0	0	0	0	0	P	N	P	N	N	N	N	N	N	N	N
91	13/08/2015	F	>3	S	0	0	0	0	0	P	P	N	N	N	N	N	N	N	P	spp.
92	13/08/2015	F	<3	S	0	0	0	0	0	P	P	N	N	N	N	N	N	N	N	N
93	13/08/2015	M	<3	S	0	0	0	0	0	N	N	N	N	N	N	N	N	N	N	N
94	13/08/2015	F	>3	S	0	0	0	0	0	P	N	N	N	N	N	N	N	N	N	N
95	23/07/2017	F	<3	S	1	<10	1	0	0	P	N	P	N	N	N	N	N	N	N	N
96	23/07/2017	F	<3	S	1	<10	1	0	0	P	N	N	N	P	N	N	N	N	N	N
97	23/07/2017	F	<3	S	1	<10	0	1	0	P	N	N	N	P	N	N	N	N	N	N
98	18/09/2017	M	<3	S	0	0	0	0	0	N	N	N	N	N	N	N	N	N	N	N
99	18/09/2017	F	<3	S	0	0	0	0	0	N	N	N	N	N	N	N	N	N	P	spp.
100	18/09/2017	F	<3	S	0	0	0	0	0	P	N	N	N	P	N	N	N	N	N	N
101	18/09/2017	M	<3	S	0	0	0	0	0	N	N	N	N	N	N	N	N	N	P	spp.
102	18/09/2017	F	<3	S	0	0	0	0	0	N	N	N	N	N	N	N	N	N	P	spp.
103	27/09/2017	F	<3	S	0	0	0	0	0	P	N	P	N	N	N	N	N	N	N	N
104	27/09/2017	F	<3	S	0	0	0	0	0	N	N	N	N	N	N	N	N	N	N	N
105	27/09/2017	M	<3	S	0	0	0	0	0	N	N	N	N	N	N	N	N	N	P	spp.
106	27/09/2017	F	<3	S	0	0	0	0	0	N	N	N	N	N	N	N	N	N	N	N
107	27/09/2017	M	<3	S	0	0	0	0	0	N	N	N	N	N	N	N	N	N	N	N
108	27/09/2017	F	>3	S	0	0	0	0	0	N	N	N	N	N	N	N	N	N	N	N
109	09/10/2017	F	<3	S	0	0	0	0	0	N	N	N	N	N	N	N	N	N	N	N
110	12/10/2017	F	>3	S	0	0	0	0	0	N	N	N	N	N	N	N	N	N	P	*T. annulata*
111	12/10/2017	F	<3	S	0	0	0	0	0	N	N	N	N	N	N	N	N	N	N	N
112	12/10/2017	F	<3	S	0	0	0	0	0	P	N	N	P	P	N	N	N	N	N	N
113	12/10/2017	M	<3	S	0	0	0	0	0	P	N	N	P	P	N	N	N	N	N	N

114	22/10/2017	F	>3	S	0	0	0	0	0	N	N	N	N	N	N	N	N	N	N	N
115	24/10/2017	F	>3	I	0	0	0	0	0	N	N	N	N	N	N	N	N	N	N	N
116	24/10/2017	F	>3	I	0	0	0	0	0	N	N	N	N	N	N	N	N	N	N	N
117	24/10/2017	F	>3	I	0	0	0	0	0	N	N	N	N	N	N	N	N	N	N	N
118	24/10/2017	F	>3	I	0	0	0	0	0	N	N	N	N	N	N	N	N	N	P	spp.
119	24/10/2017	F	<3	I	0	0	0	0	0	N	N	N	N	N	N	N	N	N	N	N
120	24/10/2017	F	<3	I	0	0	0	0	0	N	N	N	N	N	N	N	N	N	N	N
121	14/11/2018	F	>3	I	0	0	0	0	0	P	N	N	N	N	N	N	N	N	N	N
122	14/11/2018	F	>3	I	0	0	0	0	0	N	N	N	N	N	N	N	N	N	P	spp.
123	14/11/2018	F	>3	I	0	0	0	0	0	N	N	N	N	N	N	N	N	N	N	N
124	14/11/2018	F	<3	I	0	0	0	0	0	N	N	N	N	N	N	N	N	N	P	spp.
125	14/11/2018	F	<3	I	0	0	0	0	0	N	N	N	N	N	N	N	N	N	N	N
126	06/11/2017	F	<3	I	0	0	0	0	0	P	N	N	P	P	N	N	N	N	N	N
127	06/11/2017	F	>3	S	4	<10	0	0	4	P	N	N	P	P	N	N	N	N	N	N
128	06/11/2017	M	<3	S	0	0	0	0	0	N	N	N	N	N	N	N	N	N	N	N
129	06/11/2017	F	<3	S	0	0	0	0	0	P	N	N	P	P	N	N	N	N	N	N
130	06/11/2017	F	<3	S	0	0	0	0	0	N	N	N	N	N	N	N	N	N	N	N
131	19/11/2017	F	>3	S	0	0	0	0	0	N	N	N	N	N	N	N	N	N	P	spp.
132	19/11/2017	F	>3	S	0	0	0	0	0	N	N	N	N	N	N	N	N	N	P	spp.
133	19/11/2017	F	>3	S	0	0	0	0	0	N	N	N	N	N	N	N	N	N	N	N
134	19/11/2017	F	>3	S	0	0	0	0	0	N	N	N	N	N	N	N	N	N	N	N
135	27/11/2017	F	<3	I	0	0	0	0	0	P	N	N	P	N	N	N	N	N	N	N
136	27/11/2017	F	<3	I	0	0	0	0	0	N	N	N	N	N	N	N	N	N	N	N
137	27/11/2017	F	<3	I	0	0	0	0	0	N	N	N	N	N	N	N	N	N	N	N
138	27/11/2017	M	<3	I	0	0	0	0	0	N	N	N	N	N	N	N	N	N	N	N
139	27/11/2017	M	<3	I	0	0	0	0	0	N	N	N	N	N	N	N	N	N	N	N
140	27/11/2017	F	>3	I	0	0	0	0	0	N	N	N	N	N	N	N	N	N	N	N
141	27/11/2017	F	>3	I	0	0	0	0	0	N	N	N	N	N	N	N	N	N	N	N
142	27/11/2017	F	>3	I	0	0	0	0	0	N	N	N	N	N	N	N	N	N	P	spp.
143	27/11/2017	F	>3	I	0	0	0	0	0	N	N	N	N	N	N	N	N	N	N	N

144	27/11/2017	F	>3	I	0	0	0	0	0	N	N	N	N	N	N	N	N	N	N	N
145	27/11/2017	F	>3	I	0	0	0	0	0	N	N	N	N	N	N	N	N	N	N	N
146	27/11/2017	F	>3	I	0	0	0	0	0	N	N	N	N	N	N	N	N	N	N	N
147	29/11/2017	F	>3	I	0	0	0	0	0	N	N	N	N	N	N	N	N	N	N	N
148	30/11/2018	F	>3	S	1	<10	0	0	1	N	N	N	N	N	N	N	N	N	N	N
149	30/11/2017	F	>3	S	0	0	0	0	0	N	N	N	N	N	N	N	N	N	N	N
150	30/11/2017	F	>3	S	0	0	0	0	0	P	N	N	P	P	N	N	N	N	N	N
151	30/11/2017	M	>3	S	0	0	0	0	0	N	N	N	N	N	N	N	N	N	N	N
152	30/11/2017	M	>3	S	7	<10	0	4	3	N	N	N	N	N	N	N	N	N	N	N
153	30/11/2017	M	>3	S	2	<10	1	0	1	N	N	N	N	N	N	N	N	N	N	N
154	18/05/2015	F	>3	S	34	>20	20	14	0	P	N	N	N	P	N	N	N	N	N	N
155	09/07/2015	F	>3	S	0	0	0	0	0	P	N	N	N	P	N	N	N	N	N	N
156	09/05/2015	F	>3	S	6	<10	5	1	0	N	N	N	N	N	N	N	N	N	N	N
157	10/05/2015	F	>3	S	12	10_20	2	10	0	N	N	N	N	N	N	N	N	N	N	N
158	15/05/2015	F	>3	S	5	<10	4	1	0	N	N	N	N	N	N	P	N	P	N	N
159	15/05/2015	F	>3	S	1	<10	1	0	0	N	N	N	N	N	N	N	N	N	P	spp.
160	15/05/2015	F	>3	S	2	<10	2	0	0	N	N	N	N	N	N	N	N	N	P	spp.
161	15/05/2015	F	>3	S	17	10_20	15	2	0	N	N	N	N	N	N	N	N	N	N	N
162	16/05/2015	F	>3	S	3	<10	0	3	0	N	N	N	N	N	N	N	N	N	N	N
163	16/05/2015	F	>3	S	6	<10	4	2	0	P	N	N	N	P	N	N	N	N	N	N
164	16/05/2015	F	>3	S	6	<10	0	6	0	N	N	N	N	N	N	N	N	N	P	*Theileria* sp.
165	20/05/2015	F	>3	S	5	<10	3	2	0	N	N	N	N	N	N	N	N	N	N	N
166	26/05/2015	F	>3	S	4	<10	1	3	0	N	N	N	N	N	N	N	N	N	N	N
167	06/06/2015	F	>3	S	14	10_20	13	1	0	N	N	N	N	N	N	N	N	N	N	N
168	06/06/2015	F	>3	S	14	10_20	11	3	0	N	N	N	N	N	N	N	N	N	N	N
169	22/06/2015	F	>3	S	3	<10	0	3	0	N	N	N	N	N	N	N	N	N	N	N
170	22/06/2015	F	>3	S	7	<10	2	5	0	N	N	N	N	N	N	N	N	N	N	N
171	22/06/2015	F	>3	S	12	10_20	6	6	0	N	N	N	N	N	N	N	N	N	N	N
172	22/06/2015	F	<3	S	4	<10	2	2	0	N	N	N	N	N	N	N	N	N	N	N
173	22/06/2015	F	<3	S	6	<10	5	1	0	N	N	N	N	N	N	N	N	N	N	N

174	30/06/2015	F	>3	S	8	<10	3	5	0	N	N	N	N	N	N	N	N	N	N	N
175	30/06/2015	F	>3	S	21	>20	19	2	0	N	N	N	N	N	N	N	N	N	N	N
176	30/06/2015	F	>3	S	7	<10	6	1	0	N	N	N	N	N	N	N	N	N	N	N
177	30/06/2015	F	>3	S	17	10_20	14	3	0	N	N	N	N	N	N	N	N	N	N	N
178	30/06/2015	F	>3	S	17	10_20	13	4	0	N	N	N	N	N	N	N	N	N	N	N
179	30/06/2015	F	>3	S	6	<10	6	0	0	N	N	N	N	N	N	N	N	N	N	N
180	01/07/2015	F	>3	S	15	10_20	10	5	0	N	N	N	N	N	N	N	N	N	N	N
181	01/07/2015	F	>3	S	5	<10	2	3	0	N	N	N	N	N	N	N	N	N	N	N
182	01/07/2015	F	<3	S	1	<10	1	0	0	N	N	N	N	N	N	N	N	N	N	N
183	04/07/2015	F	>3	S	6	<10	6	0	0	N	N	N	N	N	N	N	N	N	N	N
184	04/07/2015	F	>3	S	4	<10	4	0	0	N	N	N	N	N	N	N	N	N	N	N
185	04/07/2015	F	>3	S	3	<10	0	3	0	N	N	N	N	N	N	N	N	N	N	N
186	04/07/2015	F	>3	S	1	<10	1	0	0	N	N	N	N	N	N	N	N	N	N	N
187	04/07/2015	F	>3	S	2	<10	2	0	0	N	N	N	N	N	N	N	N	N	N	N
188	04/07/2015	F	>3	S	7	<10	7	0	0	N	N	N	N	N	N	N	N	N	N	N
189	05/07/2015	F	>3	S	1	<10	1	0	0	P	N	N	N	P	N	N	N	N	N	N
190	28/07/2015	F	>3	S	1	<10	0	1	0	N	N	N	N	N	N	N	N	N	N	N
191	28/07/2015	F	>3	S	8	<10	0	7	1	N	N	N	N	N	N	N	N	N	N	N
192	28/07/2015	F	>3	S	4	<10	0	4	0	N	N	N	N	N	N	N	N	N	N	N
193	28/07/2015	F	>3	S	7	<10	5	2	0	N	N	N	N	N	N	N	N	N	N	N
194	28/07/2015	F	>3	S	20	10_20	4	16	0	N	N	N	N	N	N	N	N	N	N	N
195	23/07/2017	F	>3	S	1	<10	1	0	0	N	N	N	N	N	N	N	N	N	N	N
196	23/07/2017	F	>3	S	1	<10	0	1	0	P	N	P	N	N	N	N	N	N	N	N
197	23/07/2017	F	>3	S	1	<10	0	1	0	N	N	N	N	N	N	N	N	N	N	N
198	27/09/2017	F	>3	S	1	<10	0	1	0	P	N	P	N	N	N	N	N	N	N	N
199	27/09/2017	F	>3	S	4	<10	0	4	0	P	N	N	N	P	N	N	N	N	N	N
200	27/09/2017	F	>3	S	4	<10	0	4	0	N	N	N	N	N	N	N	N	N	N	N
201	12/10/2017	F	>3	S	5	<10	0	5	0	N	N	N	N	N	N	N	N	N	N	N
202	12/10/2017	F	>3	S	4	<10	0	4	0	N	N	N	N	N	N	N	N	N	N	N
203	12/10/2017	F	>3	S	1	<10	0	1	0	N	N	N	N	N	N	N	N	N	N	N

204	06/11/2017	F	>3	S	2	<10	0	0	2	N	N	N	N	N	N	P	N	N	N	N
205	06/11/2017	F	>3	S	3	<10	0	0	3	N	N	N	N	N	N	N	N	N	N	N

N°: Number, *Rh: Rhipicephalus, Hy: Hyalomma, Ix: Ixodes, A.p: Anaplasma platys. A.c: Anaplasma centrale, A.m: Anaplasma marginale. A.b: Anaplasma bovis, M.w : Mycoplasma wenyonii,* C.M.h: *'Candidatus* Mycoplasma haemobos'. N: Negative, P: Positive, F: Female, M: Male, S: Semi extensive, I: Intensive.
